# Expert Facilitated Development of an Objective Assessment Tool for Point-of-Care Ultrasound Performance in Undergraduate Medical Education

**DOI:** 10.7759/cureus.636

**Published:** 2016-06-10

**Authors:** Holly Black, Gillian Sheppard, Brian Metcalfe, Jordan Stone-McLean, Heather McCarthy, Adam Dubrowski

**Affiliations:** 1 Emergency Medicine, Memorial University of Newfoundland; 2 Faculty of Medicine, Memorial University of Newfoundland; 3 Emergency Medicine, Pediatrics, Memorial University of Newfoundland; 4 Marine Institute, Memorial University of Newfoundland

**Keywords:** ultrasound, medical education, teaching, undergraduate medical, point-of-care, assessment tool

## Abstract

Background: With the various applications of point-of-care ultrasound (PoCUS) steadily increasing, many medical schools across North America are incorporating PoCUS training into their undergraduate curricula. The Faculty of Medicine at Memorial University also intends to introduce PoCUS training into its own undergraduate medical program. The proposed approach is to introduce a PoCUS curriculum focusing on anatomy and physiology while developing cognitive and psychomotor skills that are later transferred into clinical applications. This has been the common approach taken by most undergraduate ultrasound programs in the United States. This project highlights the development and the challenges involved in creating an objective assessment tool that meets the unique needs of this proposed undergraduate ultrasound curriculum.

Methods: After a thorough review of existing literature and input from experts in PoCUS, a prototype global rating scale (GRS) and three exam-specific checklists were created by researchers. The exam-specific checklists include aorta exam, subxiphoid cardiac exam, and focused abdominal exam. A panel of 18 emergency room physicians certified in PoCUS were recruited to evaluate the GRS and three checklists. This was accomplished using a modified Delphi technique. The items were rated on a 5-point Likert scale. If an item received a mean score of less than 4, it was deemed unimportant for the assessment of PoCUS performance in undergraduate medical learners and was excluded. Experts were also encouraged to provide comments and suggest further items to be added to the GRS or checklists. Items were modified according to these comments. All of the edits were then sent back to the experts for revisions.

Results: A consensus was achieved after three rounds of surveys, with the final GRS containing nine items. The final aorta checklist contained nine items, and the subxiphoid cardiac and focused abdominal checklists each contained 11 items.

Conclusion: By using a modified Delphi technique, we developed a single GRS and three checklists. A panel of independent PoCUS practitioners supports the content validity of these tools. Research is currently ongoing to evaluate their validity for assessing PoCUS competency in undergraduate medical students.

## Introduction

In the last two decades the medical community has seen a steady increase in the use of point-of-care ultrasound (PoCUS) [1]. PoCUS, or use of ultrasound at a patient’s bedside, is now part of the daily practice of many physicians in various specialties. The wide acceptance of PoCUS is largely attributable to the versatility of ultrasound imaging and the advent of smaller, cheaper ultrasound machines that provide high quality images [1-2]. PoCUS has become both an adjunct to the physical exam as well as an important tool used in many procedures [1, 3-7]. Visualization of vital internal structures using ultrasound can narrow the clinician’s differential diagnosis and improve diagnostic accuracy [8]. Ultrasound guided procedures include, but are not limited to, central venous catheter placement, regional anesthesia, thoracentesis, and paracentesis [1, 9]. A review of the literature shows the body of research regarding PoCUS is rapidly growing, expanding the list of PoCUS related procedures and exams. When used appropriately these procedures and exams can ultimately lead to superior patient care and safety [1, 10].

In addition to its versatility, safety, and overall patient benefits, it has also been shown that PoCUS can safely be taught to medical students and novice examiners [8, 11-15].  Therefore, it is not surprising that a number of medical programs are piloting and incorporating PoCUS into their curricula [5, 16-21]. Medical schools need to develop PoCUS programs if they are to ensure their students remain current with modern medical advances and are adequately prepared to face the changing demands of clinical practice [22-23]. Memorial University’s Faculty of Medicine recognizes this need and endeavors to train competent and knowledgeable generalists who are able to meet these demands [Metcalfe et al., 2014; http://www.wcume.org/wp-content/uploads/2015/09/v6-OHSU_14_01-WC-Ultrasound-Conference_Guide-Book_V6.pdf].

**Figure 1:**
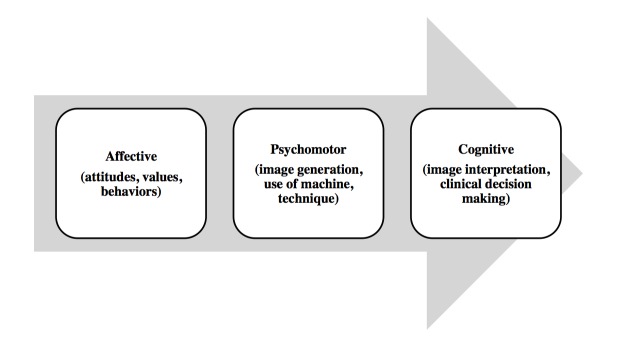
Skills Translation Model adapted by the Memorial University’s approach to an undergraduate PoCUS curriculum.

Our approach is to develop and introduce an undergraduate PoCUS curriculum focusing on anatomy and physiology while developing cognitive and psychomotor skills that can later be transferred into clinical applications. This has been the common approach taken by most undergraduate ultrasound programs in the United States [24]. Figure 1* *demonstrates the educational theory behind Memorial University’s approach to developing and introducing an undergraduate PoCUS curriculum. This educational theory is based on Bloom’s three domains of learning [25]. It presumes that undergraduate PoCUS learners acquire the attitudes and physical proficiencies to perform ultrasound first, followed later in their medical school career by the ability to “put it all together” in the making of clinical decisions.

Like any clinical skill, the addition of PoCUS to medical school curricula necessitates evaluation. We must be able to assess the level of competence on the part of the practitioner since the inappropriate use of PoCUS can be dangerous [26]. Traditional assessment of clinicians participating in PoCUS training courses includes observation with subsequent written and visual exams. To assess medical students in the same manner as experienced clinicians may not be appropriate [27]. Accordingly, undergraduate medical education requires its own unique form of assessment. Although assessment tools have been designed for practicing clinicians [28], and milestones for curricula development have been suggested [24], we are unaware of any existing assessment created specifically for undergraduate learners. We, therefore, set out to create an assessment tool that would fit within our educational framework and also meet the unique needs of our proposed undergraduate program. Our tool is designed with the intention of observing and assessing medical students over time. This observation would occur both in pre-clerkship, when they have minimal knowledge of PoCUS and its applications, and in clerkship, when they should be better able to understand and appreciate the many clinical applications of PoCUS.

## Materials and methods

A modified Delphi technique was used to obtain expert consensus on items to be included in the assessment tool. The Delphi technique requires a panel of experts to complete several rounds of an opinion-eliciting survey. The responses to the survey are then collected, analyzed, summarized, and redistributed to the experts in the form of a new survey. Multiple iterations are used to achieve consensus [29]. This method encourages debate while maintaining anonymity, lessening the impact of strong opinions and personalities on final consensus formation [30].

A non-probability sampling technique known as purposive sampling was used to select the expert panel to which the survey would be distributed. We initially administered the survey to a group of highly trained specialists practicing in a variety of areas including emergency medicine, anesthesia, intensive care, obstetrics/gynecology, otolaryngology, and radiology. The initial administration of the survey was used to analyze the feasibility of the study, specifically as it related to participant recruitment/survey administration and sample/panelist selection. Some of these participants indicated their clinical use of ultrasound is highly specialized. As a result, they did not feel they could comment on how to assess learners who would practice PoCUS as generalists. For example, an intensivist said, “I’m not sure I am best placed to respond to this survey. My skill set is not as comprehensive as ER docs.”

Based on this feedback, the participant inclusion criteria was revised, limiting participants to local emergency physicians as they were thought to be best equipped to aid in the development of a tool for learners who will be trained as generalists in PoCUS. These participants were certified with the Canadian Emergency Ultrasound Society as Independent Practitioners and Master Instructors.

The survey was then redistributed to these experts who were asked to give their opinion on a “Point of Care Ultrasound Assessment Tool for Undergraduate Medical Education” comprised of a Global Rating Scale (GRS) and three anatomically specific checklists (aorta, focused abdominal exam, and cardiac). Systematic reviews have shown checklists and GRS’s have differing strengths and weaknesses [31], making the simultaneous use of both GRS and checklists valuable. The GRS was modified from the original Objective Structured Assessment of Technical Skills (OSATS) to include skills specific to ultrasound. Both the GRS [32] and the OSATS method of assessment have been found to be valid and reliable [32-36].

Each expert was asked to score each GRS item and each checklist item as “yes” for inclusion or “no” for exclusion. The experts were then asked to rate these same items on a Likert scale ranging from 1-5. As in other published studies, the items that had a mean score of greater than 4 and standard deviation (SD) ≤.5 were deemed important for the assessment of PoCUS performance in undergraduate medical learners [37]. If an item received a mean score of less than 4, it was deemed unimportant for this assessment and was excluded. In addition, we encouraged the experts to provide comments and suggest additional items that might be included. Items were modified accordingly. Items that met inclusion criteria, but could be improved with adaptation according to experts’ comments, were also modified. If no comments were made but the item required revision, key informants were consulted. All changes and additions were analyzed, summarized, and then returned to the experts in subsequent rounds of the survey. When the participants suggested no additional items or significant changes, the results were compiled, and the final GRS and checklists were created.

## Results

The final GRS and checklists were developed after three rounds of surveys.

All 18 invited reviewers completed the first round of the survey. Based on the Delphi method criteria, only three of the 11 initial GRS items were accepted by the reviewers--image interpretation, knowledge of procedure (if applicable), and overall performance. The other eight items were either rejected or modified based on the reviewers’ comments. The expert panel also suggested two new items be added to the GRS--documentation of ultrasound image, and demonstrates understanding of personal and technical limitations.

Similarly, based on the Delphi method criteria, 11 of the original 23 checklist items were accepted for each of their final respective checklists. The item 'landmarks xiphoid process' was rejected from the focused abdominal ultrasound checklist based on the Delphi criteria. The researchers revised the remaining 11 checklist items for the next round of the survey based on the reviewers’ comments. The new items suggested by the reviewers were: differentiates aorta from IVC, identifies left and right ventricle, scans pelvis, identifies Pouch of Douglas or recto-vesical pouch and presence of fluid, and demonstrates techniques for dealing with rib shadows.

All 18 reviewers completed the second iteration of the survey. In round two, they reviewed the six updated and the two new GRS items. Two items--image optimization (probe choice, optimization of gain and depth) and probe technique--were accepted for inclusion in the final version of the GRS. The panel rejected two items: documentation of ultrasound image, and demonstrates understanding of personal and technical limitations. The remaining four items were sent back to researchers to be revised.

In round two, the expert panel reviewed a combined total of 15 revised and new checklist items. Five of the revised checklist items were accepted for inclusion in each of their final respective checklists (obtains verbal consent for bedside ultrasound when possible, ensures patient is in a comfortable position and is draped appropriately, scans aorta from diaphragm to bifurcation, accurately measures aorta by measuring outside wall to outside wall, identifies the apex and septum). The reviewers rejected three items (scans pelvis, identifies Pouch of Douglas or recto-vesical pouch, and presence of fluid). The remaining checklist items were sent back to researchers for further revision based on the reviewers’ comments.

**Figure 2:**
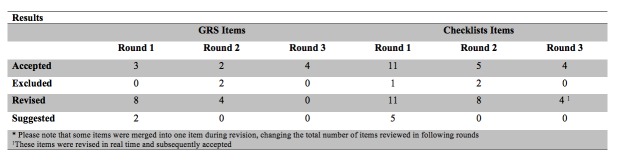
Results: Number of items in each of the tools that were accepted, excluded, revised, and alternations suggested per round.

**Figure 3:**
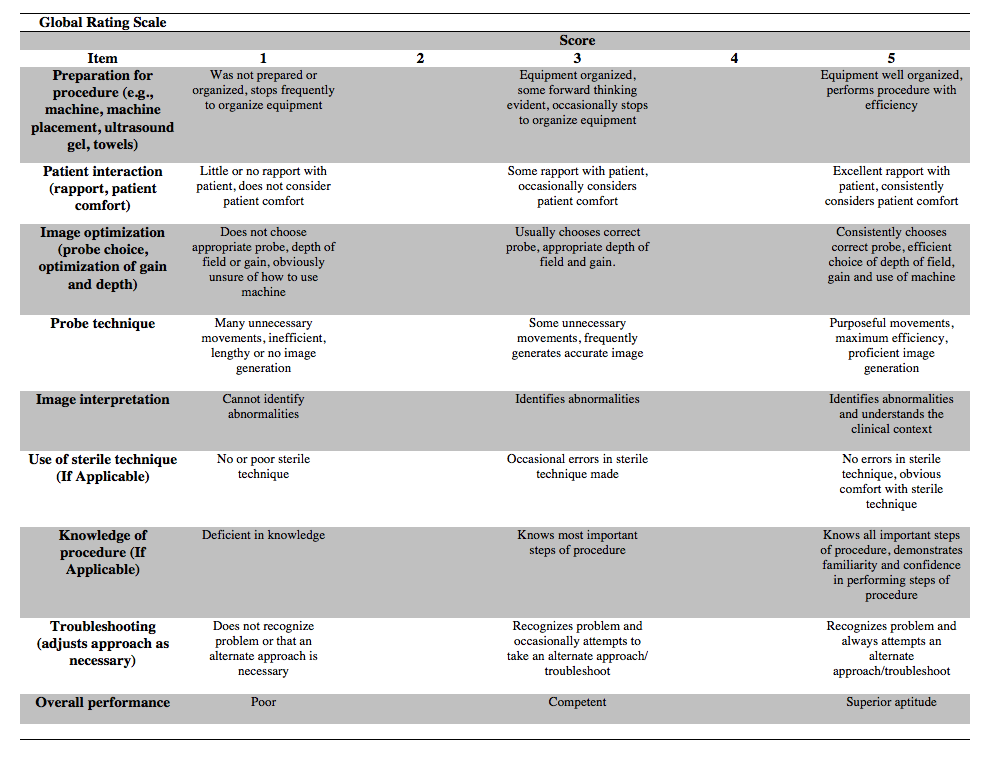
Final Global Rating Scales.

**Figure 4:**
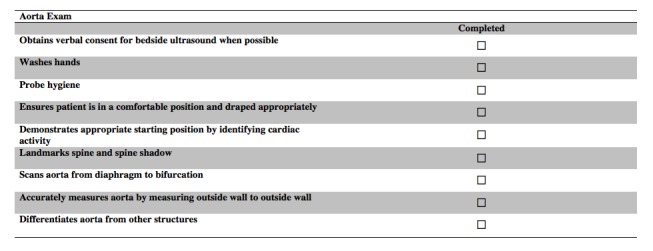
Final Aorta Exam Checklist.

**Figure 5:**
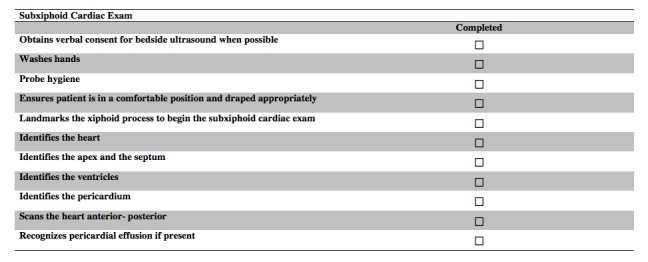
Final Subxiphoid Cardiac Exam Checklist.

**Figure 6:**
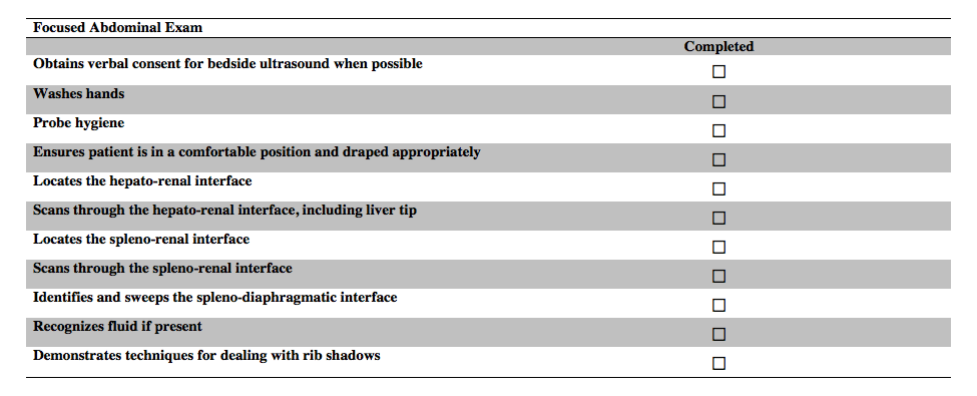
Final Focused Abdominal Exam Checklist.

In round three, nine of the invited reviewers completed the third and last iteration of the survey. This round was completed in real time, face-to-face. They reviewed four GRS items (preparation for procedure--machine, machine placement, gel, towels; patient interaction--rapport, patient comfort; use of sterile technique--if applicable; troubleshooting--adjusts approach as necessary), and eight checklist items (washes hands before performing ultrasound, demonstrates appropriate starting position by identifying cardiac activity, landmarks spine and spine shadow, differentiates aorta from IVC and other vascular structures, landmarks the xiphoid process to begin the subxiphoid cardiac exam, identifies left and right ventricle of the heart, scans and sweeps splenodiaphragmatic interface, demonstrates techniques for dealing with rib shadows) from the previous round. The reviewers’ comments were used to modify items in real time. The reviewers accepted all of the items once they were modified according to the comments made in the session. For clarity, each round and the number accepted, excluded, revised, and suggested items are shown in Figure 2*.* The final GRS, aorta, subxiphoid cardiac, and focused abdominal ultrasound checklists are shown below. (Figures 3-6)

## Discussion

The purpose of this project was to create an objective assessment tool that will meet the needs of Memorial University Faculty of Medicine’s proposed PoCUS curriculum. The evaluation of ultrasound skills must include objective, reliable, and validated assessment tools, as they are necessary to ensure that a standard, general level of competence is attained [1]. Given that some undergraduate learning objectives may differ from those of postgraduate and clinician training courses, this proposed PoCUS undergraduate program requires a unique form of assessment.

The Faculty of Medicine at Memorial University trains undergraduate students with the objective of preparing them as generalists, ready to enter whichever residency program they choose. As a result, any newly introduced PoCUS program must also align with this key principle. The knowledge base, clinical competency, and needs of postgraduate learners differ from those in undergraduate medicine. Undergraduate medical students cannot be expected to study and be assessed using a PoCUS curriculum that is ultimately aimed at a different level and type of learner. Interestingly, this became evident when our survey was first administered to a variety of specialists. While we initially thought that recruiting specialists for the study would result in the development of a more comprehensive assessment tool, the specialists felt uncomfortable developing a tool that would be used to assess generalists. Given the broad scope of practice and generalist approach used in emergency medicine, we decided that emergency medicine physicians would be best suited to comment on a tool meant to assess learners who would be trained as generalists.

The proposed PoCUS program at Memorial University envisions undergraduate students learning anatomy/physiology and physical exam skills while concurrently gaining the skills necessary to perform PoCUS. Initially, students will learn the general principles of PoCUS, such as ultrasound physics and probe technique, while simultaneously gaining anatomical knowledge and clinical skills. By introducing these skills early, students will have more opportunity to practice their PoCUS skills, and faculty will have more time to assess them. This ensures they are competent and qualified to practice at the end of medical school. This proposed curriculum and accompanying assessment tool strengthen the effectiveness of the assessment of PoCUS skills in two ways. First, the assessment is more effective if done over time by different faculty [38]. Second, more consistent feedback is gained when using a tool designed specifically for a skill like PoCUS [38]. Learners in this proposed undergraduate curriculum will have far more time for structured and consistent assessment compared to their post-graduate counterparts. It is our belief that better assessment leads to better skills.

Objective tools like the one developed here enhance the learning process by facilitating constructive feedback based on each specific item and marking progression over time [39]. Some may argue that this is impractical and could make learning and the subsequent assessment of anatomy/physiology and PoCUS more difficult. However, in other domains of knowledge acquisition, such as critical thinking, evidence suggests that incorporating critical thinking into existing subjects may be advantageous as compared to having a separate course on critical thinking [40]. Although further research is required to determine if the same principles apply to teaching ultrasound skills across curriculum and existing courses, at this stage we speculate that if not beneficial, at least this approach is not harmful. Furthermore, this tool was modified from previously existing assessment tools that have been shown to be valid and reliable [32-34]. As well, it can be used in both the simulation lab and the clinical area [39, 41], making this form of assessment both effective and efficient.

Our research team acknowledges several limitations. This project was potentially impacted by both sample size and composition. Despite the growing popularity of PoCUS, there are a limited number of certified, independent practitioners at our site. Furthermore, these participants have for the most part all undergone similar training by a limited number of instructors. A larger, more diverse sample size may have provided more varied responses. Moreover, as a result of the small sample size, items were rounded down and accepted with a standard deviation of .51. Without this alteration, seemingly important items that received only “important” or “very important” on the Likert scale would not have been accepted for inclusion in the final assessment tool.

Additionally, the Delphi technique was modified so that participants fully understood the primary objective of the project, which was to create an assessment for use in an undergraduate curriculum. Educational methods used for novices can be ineffective in training experts. This is known as expertise reversal effect [42]. Furthermore, all assessment methods are not always appropriate for differing levels of learners [27]. As a result, we thought it imperative to ensure that the experts helping to create the tool thought about the assessment of PoCUS at the undergraduate level and did not solely think about how they themselves as experts would be assessed. Accordingly, as a means of stressing this point, the third round of the survey was conducted face-to-face rather than online. Every effort was made to avoid biasing the panel. Lastly, caution must be taken when using an opinion-based assessment tool [36]; however, we believe this issue can be addressed in future projects by testing the validity and reliability of this tool using independent raters.

## Conclusions

Using a modified Delphi technique, we were able to create an objective assessment tool for undergraduate PoCUS learners at Memorial University. A panel of expert PoCUS practitioners supported the content validity of this tool. Research is currently ongoing to evaluate this tool’s validity in assessing PoCUS competency in undergraduate medical students. At a time when many medical schools are changing their curricula to coincide with changing national exams, this technique can be modified and used to create further objective assessment tools.
